# Management of Malignant Pleural Effusion with ASEPT® Pleural Catheter: Quality of Life, Feasibility, and Patient Satisfaction

**DOI:** 10.1155/2016/4273480

**Published:** 2016-12-15

**Authors:** Inderdeep Dhaliwal, Masoud Mahdavian, Shabnam Asghari, Benson Chun To Wong, Rosalie Labelle, Kayvan Amjadi

**Affiliations:** ^1^Schulich School of Medicine, Western University, Victoria Hospital, London Health Sciences Centre, London, ON, Canada; ^2^Division of Respirology, Faculty of Medicine, Memorial University of Newfoundland (MUN), St. John's, NL, Canada; ^3^Discipline of Family Medicine, Health Sciences Centre, Memorial University of Newfoundland (MUN), St. John's, NL, Canada; ^4^Division of Respirology, University of Ottawa, The Ottawa Hospital, Ottawa, ON, Canada; ^5^Department of Medicine, Division of Interventional Pulmonology, The Ottawa Hospital, Ottawa, ON, Canada; ^6^Division of Interventional Pulmonology, University of Ottawa, The Ottawa Hospital, Ottawa, ON, Canada

## Abstract

*Objective*. The PleurX® IPC system has been used extensively in the past. Over time, management of MPE with the PleurX system can be costly. The new ASEPT pleural catheter, through advantages in design, may ultimately show cost savings. The primary outcome of this study was to evaluate safety and efficacy of the ASEPT system.* Method*. This single centre, prospective study enrolled 50 patients with MPE, who were followed for as long as they were alive with a catheter. Quality of Life (QoL) was assessed before, at 2 weeks, and 6 weeks after ASEPT catheter insertion using the EORTC QLQ-C30 and LC13 questionnaires. Ease of catheter use and complications were reported by physician and community nurses.* Results*. 50 patients with MPE with a mean age of 64.5 ± 1.9, BDI of 2.8 ± 0.9, and ECOG score of 3.0 ± 0.7 were recruited. No immediate or long-term complications were reported during the study period. Compared to precatheter insertion, global health status (−18, *p* < 0.001), QLQ-C30 dyspnea (−39, *p* < 0.00001), and LC13 dyspnea (−11, *p* < 0.0005) significantly improved at 2 and 6 weeks after intervention. Provider surveys indicated favourable ease of use.* Conclusion*. The new ASEPT catheter offers a safe and effective option for the management of MPE.

## 1. Introduction

Malignant pleural effusions (MPEs) occur in 25–50% of malignancies, and the majority (75%) of cases are symptomatic (dyspnea 57%, cough 43%, weight loss 32%, and chest pain 26%) [1]. In general, MPE portends a poor prognosis yielding a 30-day mortality of 29–50% and an average survival of 3–9 months [2, 3]. Therefore, palliative management for MPE aims at alleviation of related symptoms. Tube thoracostomy with chemical pleurodesis, chronic indwelling pleural catheters (IPCs), and thoracoscopy with pleurodesis are currently the main therapeutic modalities offered at various health care facilities. The British Thoracic Society (BTS) guidelines support all options in appropriate patient population [2]. IPCs provide a unique advantage to other modalities; they can be inserted in an ambulatory setting which reduces hospitalization, increases time spent at home, and requires fewer subsequent pleural procedures to obtain symptom palliation [3]. Within our region, all patients with IPC receive home nursing care for interrupted drainage and dressing changes. Depending on survival and drainage frequency, this can generate significant cost to the system; thus cost saving strategies are imperative.

Many authors have demonstrated successful management of MPE with PleurX catheter (Cardinal Biomedical) [4–9]. PleurX catheter has an extended external length of approximately 28 cm, which complicates dressing changes and maintenance of sterile field during drainage sessions. Furthermore, a large dressing is required for adequate coverage of the catheter and in order to provide a water proof seal, so that the patient can take a shower. This causes dressing related skin complications, specifically in individuals with fragile, dry skin secondary to chemotherapy. In addition, its unique one-way valve is not repairable or replaceable. Thus, if the valve becomes dysfunctional, the entire catheter needs to be replaced, resulting in procedure related discomfort for the patients. The PleurX system requires use of a proprietary drainage catheter combined with a drainage bottle which limits accessibility to pleural fluid. Previously, in North America, the PleurX catheter was the only commercially available small bore catheter designed specifically for long-term drainage of malignant pleural effusion. The recently introduced ASEPT pleural catheter (PFM Medical USA Inc., Carlsbad, CA, USA) is designed with a one-way valve system attached to a luer lock connector, modifiable external length, and a repair kit. Luer lock access enables providers to use cheap, readily available drainage lines and bottles to facilitate interrupted drainage. Repair of the damaged catheter port can mitigate the need to replace the entire catheter (as required currently with the PleurX catheters). In addition, a smaller external length could allow for smaller dressing use which may provide superior patient comfort. To this effect, the ASEPT catheter and drainage bottles may offer significant patient preference and cost saving over the PleurX system. The ASEPT pleural catheter has been FDA and Health Canada approved (since 2009 and 2011, respectively) for the treatment of pleural effusions.

The aim of this study was to evaluate the safety and effectiveness of the ASEPT catheter in managing malignant pleural effusions in terms of patients' self-rated Quality of Life (QoL), its ease of use, the incidence of complications, and levels of health care provider satisfaction. Results from this study will determine if a larger, randomized trial is warranted to compare the current standard of care PleurX to the ASEPT pleural catheter at our institution and to conduct a cost analysis. The primary outcome of this study was to assess patients' self-rated Quality of Life (using the EORTC QLQ-C30 and LC13 questionnaires) as well as studying physician and nurses reported ease of use with the ASEPT pleural catheter, in an inpatient and outpatient setting among patients with MPEs. All nursing staff were trained and comfortable with the use of both PleurX and ASEPT catheter products. The secondary outcomes were to assess the complications related to the use of ASEPT pleural catheter, in particular with regard to the longevity of its one-way valve as well as hospital and home care nursing staff satisfaction with the device.

## 2. Methods

We designed a nonblinded longitudinal study to address the aim of this study and its outcome. Patients were followed for as long as they had an indwelling catheter or until death. However, for the purpose of this study, collection and analysis of Quality of Life data were limited to 42 days after insertion of the catheter. This study was approved by the Ottawa Health Science Network Research Ethics Board (protocol number 2011963-01H). This study was conducted at the Ottawa Hospital, General Campus, between January 1st, 2014, and February 1st 2015, and enrolled patients presenting to the Chronic Ascites and Recurrent Effusion (CARE) Clinic with symptomatic/recurrent MPEs who were scheduled to receive an IPC. Patients with age ≥18-year-old, symptomatic (dyspnea with BDI < 6) moderate sized effusion (>1/3 hemothorax), free flowing MPE, and life expectancy greater than 2 months were included in this study. Eligible patients were asked to provide consent for use of data collected from their routine medical assessments following insertion of the ASEPT catheter. Information was gathered at baseline and 2 weeks and 6 weeks following catheter insertion (Table 3). Patients who had the ASEPT catheter removed prior to the completion of the study were asked to complete the questionnaires at that time of catheter removal. Hospital and home care nurses were asked to complete questionnaires indicating the ease of use and their level of satisfaction with the ASEPT catheter at the same time intervals. Similarly, the study physician completed a questionnaire assessing ease of use of the catheter following its insertion and removal.

Quality of Life assessment at baseline, 2 weeks, and 6 weeks was performed using well-validated European Organization for Research and Treatment of Cancer Quality of Life Questionnaire (EORTC QLQ30) and Lung Cancer (LC-13) specific Quality of Life scores [1, 10]. Dyspnea measurement was performed at the same intervals using Baseline Dyspnea Index (BDI) on initial visit followed by Transitional Dyspnea Index (TDI) to evaluate for improvement [11].

Sample size calculations were performed with a paired *t*-test, using an alpha of 0.05, an estimated change of at least 10 points in the EORTC score, and a beta of 0.8 with SD of 20. These factors resulted in a required sample size of 35; however we increased this to 50 patients to account for subject mortality and losses to follow-up.

Baseline and follow-up data were entered in a case report form and all questionnaire data were entered by each subject on the questionnaire itself. All data were anonymized and transferred to a statistical software spreadsheet. The primary outcome measures for this study were (i) pre- and post-ASEPT catheter insertion EORTC QLQ-C30 and LC13 scores and (ii) reported ease of use. Secondary outcome measures were (i) the incidence of complications and (ii) pre-and post-ASEPT catheter levels of satisfaction. Pre- and post-ASEPT catheter EORTC scores and levels of satisfaction were analyzed using paired *t*-tests. The incidence rate (with 95% confidence intervals) was used to describe complications among participants.

## 3. Results

**Figure 1:**
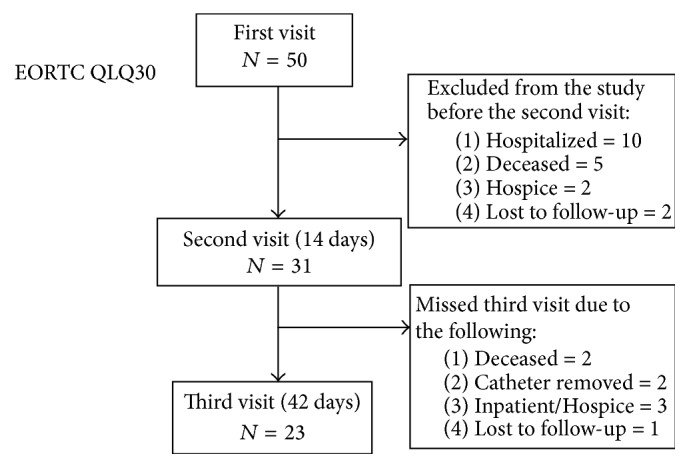
Course of the study.

There were 50 patients with MPE who were recruited for ASEPT pleural catheter insertion. The study population had an average age of 64.5-year-old (95% CI: 60.7–68.2) with 62% female (Table 1). The main causes of MPE were lung cancer (30%) and breast cancer (26%) followed by ovarian cancer (12%). Insertions of ASEPT pleural catheter were feasible in 100% of cases. On average 1.4 ± 0.3 L of pleural fluid was drained upon insertion of the catheter. Drainage was primarily stopped upon complete evacuation of the pleural space (56%), development of pernicious cough (32%), or chest discomfort (12%). Study follow-up at 2 and 6 weeks is outlined in Figure 1. There were 26 patients who had IPC removed during the study period with a mean and median duration of 92.3 ± 15.6 and 73 days, respectively. No complications were reported during the study period. However, 2 catheters required replacement of the valve due to damage. This occurred as a result of a nursing error leading to the access of the ASEPT catheter's valve with a proprietary PleurX drainage kit. The damaged valve was easily replaced without any negative health consequences to the patient. More specifically, there was no leakage of effusion from or suction of air into the chest cavity.

The patients were initially symptomatic with a mean BDI of 2.7 ± 0.99 and mean ECOG 3 ± 0.7. Dyspnea significantly improved after ASEPT pleural catheter insertions (mean TDI: 6.2 ± 0.2, 95% CI: 5.8–6.7).

**Figure 2:**
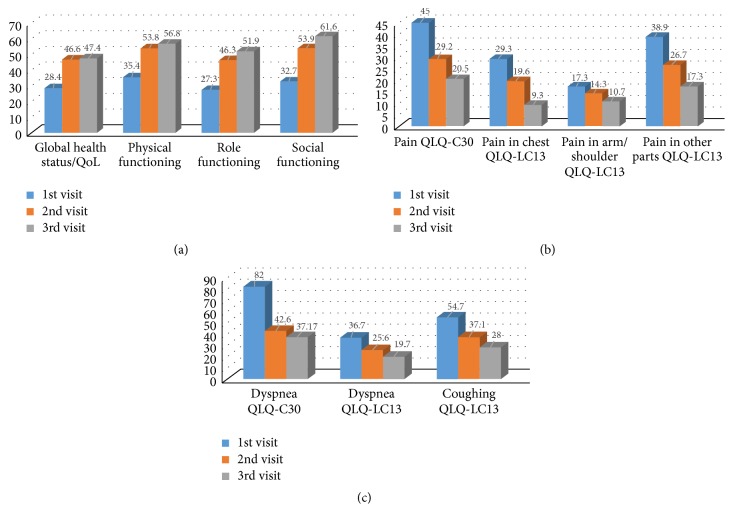
(a) Comparison of global health status/Quality of Life and its functional scales in 1st visit and after ASEPT pleural catheter insertion (2nd and 3rd visits) according to the EORTC QLQ-C30. (b) Comparison of pain scores in 1st visit and after ASEPT pleural catheter insertion (2nd and 3rd visits) according to the EORTC QLQ-C30 and QLQ-LC13. (c) Comparison of dyspnea and coughing scores in 1st visit and after ASEPT pleural catheter insertion (2nd and 3rd visits) according to the EORTC QLQ-C30 and QLQ-LC13.

The assessment of global health status/Quality of Life and its domains (functional and symptom scales) by EORTC QLQ-C30 and LC13 demonstrated significant clinical and statistical improvements at 2 and 6 weeks after intervention which was maintained during the study period (Table 2 and Figure 2). All domains impacting QoL improved by greater than 10 points with the exception of cognitive functioning at 2 weeks which had a mean change of 8.9 points. Mean change in global QoL improvement was 18.2 (*p* value < 0.001) and was maintained throughout the study period. Significant improvement in dyspnea was noted after insertion of catheter (mean QLQ-C30 dyspnea score improved by 39 points; *p* value < 0.00001 and mean QLQ-LC13 dyspnea score changed by 11 points, *p* value < 0.0005) (Table 2 and Figure 2).

Finally, nursing feedback on the ASEPT system was positive. The shorter external catheter has been found to be more desirable by our home care nurses. They report satisfactory coverage of the catheter with a smaller dressing that reduces the skin's surface area in contact with the adhesive tape, minimizing skin irritation. Furthermore, they find that maintenance of a sterile field during drainage sessions was easier with a shorter catheter. With longer catheters there is need to fold the tubing into a pretzel shape which would then be held in place until the larger, water proof, self-adhesive tape can be applied. Depending on the position of the catheter, patient's body habitus, and their position during dressing changes (seated or supine), the process can be quite challenging and nurses often claim they require “a third hand.” Upon removal of the protective dressings, the longer catheters have the tendency to unfold out of the sterile field providing an overall frustrating nursing experience when it comes to caring for the catheters. Simply, shortening the external length of the catheter to approximately 4 cm tends to eliminate these issues.

## 4. Discussion

To our knowledge, there are no prior studies evaluating ASEPT pleural catheters for management of MPE, and therefore, we are unable to compare our data to previously published reports. However, our reported improvements in Quality of Life appear to be comparable to previously reported studies using PleurX catheters [12, 13]. Sabur et al. reported a significant improvement in global health status and dyspnea at 2 weeks after IPCs insertion with mean change in QLQ-C30 of −32.4 (*n* = 68, *p* < 0.001) and dyspnea QLQ-LC13 mean change of −20.4, (*n* = 56, *p* < 0.001) [12]. Although we did not design the study as a comparison, the QoL benefit in our study was similar to the reported literature (QLQ-C30 dyspnea score of −39 and LC13 dyspnea score of −11). Lorenzo et al. showed that, in a group of 51 Spanish out-patients with recurrent MPE, IPC had significantly improved EORTC QLQ symptoms scores; however, it failed to demonstrate a statistically significant improvement in global health status and functional scales at 30 and 60 days [13]. In contrast, using the ASEPT catheter, global and physical QoL were significantly improved throughout the study duration. The ASEPT catheter may produce similar if not superior efficacy to the reported literature on the PleurX system, although this was not within the scope of this study [12, 13]. There were no safety issues surrounding the ASEPT catheter apart from nursing error with incorrect accessing of the luer lock that led to damage of the one-way valve. This error may be mitigated with dedicated teaching of home care staff.

Several recent cost analysis studies suggest IPC to be the most cost-effective therapeutic option for the management of MPE [14, 15]. In the United States, Shafiq and colleagues found that IPC management was the most cost-effective alternative to repeated thoracentesis in management of MPE [14]. This analysis was done to 6 months, which was deemed to be the average survival of patients with MPE [14]. Cost-effectiveness of IPC for patients surviving longer than 6 months is not available but can be expected to be more costly. In a Spanish cohort, TPC was again found to be a cost-effective strategy for MPE management although there was no alternative comparison. Outpatient insertion and survival less than 3 months were factors associated with lower costs [15]. These studies were conducted using the PleurX system; we believe that the use of ASEPT pleural catheters can further reduce the cost of ongoing MPE management for patients with longer survival. For our region, cost of PleurX drainage bottle is CDN $80, while ASEPT bottles are CDN $20. In addition, it is easily possible to repair the ASEPT valve system, while the PleurX often requires reinsertion. We hope to prospectively study cost-effectiveness between the two catheter systems in the future.

The primary goal of care in patients with refractory MPE is alleviation of associated symptoms [4]. IPCs play an important role as a therapeutic modality for this patient population, facilitating outpatient care [3]. This prospective study is the first formal evaluation of the ASEPT pleural catheter for the management of MPE, demonstrating a significant improvement in patients' symptoms and QoL. These improvements were sustained during the study period of 6 weeks.

Although we are encouraged by the outcome of our study and have included ASEPT catheters in our inventory for management of refractory MPE, we recognize some limitations in our study. We are aware that this is a relatively small study and thus, we are unable to comment on the longevity and integrity of the catheter's valve beyond our study's period. Although to date we have not experienced any valve related complications, we are reassured by the fact that a repair kit is available should it become necessary. With ongoing use of the ASEPT catheter, we are continuing to gather information with regard to its structural durability and long-term safety. Furthermore, we did not engage in a comparative study between the two existing catheters and thus are unable to report superiority of one catheter over the other nor can we suggest that the shorter external length of the catheter is superior to the longer one. However, we are encouraged by our low infection rate (none), absence of dressing related skin issues, and excellent nursing feedback supporting the use of a shorter catheter. In addition, due to the devastating nature of metastatic cancer we had a significant number of deaths and patients lost to follow-up. This highlights our limited capacity to predict survival in patients with terminal malignancy; superior assessment tools are necessary to better design prospective studies in this patient population. Our study does suggest that the ASEPT catheter can be used as an alternative product with potential cost saving and at no apparent increased risk to our patients with MPE. The single centre nature of the study and the lack of control group are additional weaknesses that we acknowledge. We plan on conducting a multicentre Canadian study in the near future.

## 5. Conclusion

This prospective study demonstrates that management of refractory MPE with ASEPT pleural catheters is feasible and associated with a statistically significant improvement in dyspnea and overall Quality of Life as measured by the EORTC QLQ-C30 and QLQ-LC13. Further RCT investigations are suggested to assess its safety and efficacy compared to the PleurX system and to conduct a cost analysis.

## Figures and Tables

**Table 1:** Demographic information and clinical variables of the study.

Item	Result
Age	64.5 ± 1.9
Gender (female)	31 (62%)
BDI	2.78 ± 0.14
TDI	6.24 ± 0.2
Cause of MPE	
Lung cancers	15 (30%)
Breast cancers	13 (26%)
GU cancers	7 (14%)
GI cancers	4 (8%)
Lymphoma	3 (6%)
Other cancers	8 (16%)
Side	
Left	23 (46%)
Right	27 (54%)
Limiting factor to stop draining pleural fluid after insertion of ASEPT catheter	
Cough	16 (32%)
Pain	6 (12%)
No more fluid	28 (56%)

BDI: Baseline Dyspnea Index, TDI: Transitional Dyspnea Index, and MPE: Malignant Pleural Effusion.

**Table 2:** Quality of life domains in different visits.

Item	1st visit (*N*: 50)	2nd visit (*N*: 31)		3rd visit (*N*: 23)		4th visit (*N*: 4)	
	Mean ± SE	Mean ± SE	Δ^*∗*^	Mean ± SE	Δ^*∗*^	Mean ± SE	Δ^*∗*^
*EORTC QLQ-C30*							
Global health status/QoL	28.4 ± 3.2	46.6 ± 4.2	−18.2	47.4 ± 4.9	−19	81.3 ± 9.2	−52.9
Functional scales							
Physical functioning	35.4 ± 3.5	53.8 ± 4.47	−18.4	56.8 ± 5.5	−21.4	85.8 ± 7.2	
Role functioning	27.3 ± 4.6	46.3 ± 5.7	−19	51.9 ± 6.5	−24.6	87.5 ± 12.5	
Cognitive functioning	67.7 ± 4.5	76.6 ± 4.3	−8.9	79.2 ± 3.6	−11.5	91.6 ± 8.3	
Social functioning	32.7 ± 4.6	53.9 ± 5.7	−21.2	61.6 ± 6.9	−28.9	83.3 ± 6.8	
Symptoms scales							
Pain	45 ± 5.2	29.2 ± 4.4	+15.8	20.5 ± 6.0	+24.5	16.7 ± 6.8	
Dyspnea	82 ± 3.6	42.6 ± 5.7	+39.4	37.17 ± 6.7	+44.8	16.7 ± 9.6	
Insomnia	59.9 ± 4.9	43.5 ± 5.7	+16.4	35.9 ± 6.9	+24	25 ± 15.9	
Financial difficulties	28.7 ± 5.3	17.6 ± 4.8	+11.1	12.8 ± 5.3	+15.9	0 ± 0	

*QLQ-LC13*							
Dyspnea	36.72 ± 2.3	25.6 ± 3.3	+11.1	19.7 ± 3.7	+17		
Coughing	54.7 ± 4.5	37.1 ± 4.2	+17.6	28 ± 5.3	+ 26.7		
Hemoptysis	4.7 ± 1.9	0 ± 0		0 ± 0	+4.7		
Pain in chest	29.3 ± 4.7	19.6 ± 4.5	+9.7	9.3 ± 3.6	+20		
Pain in arm or shoulder	17.3 ± 3.7	14.3 ± 3.9	+3.0	10.7 ± 4.1	+6.6		
Pain in other parts	38.9 ± 5.6	26.7 ± 5.2	+12.2	17.3 ± 5.8	+24.5		

^*∗*^Compared to the 1st visit.

**Table 3:** Visit schedule.

Study day (±2 days)	Visit 1	Visit 2	Visit 3
	Day 1	Day 14	Day 42
Patient consent	X		
Medical history^a^	X	X	X
Physical exam^a^	X	X	X
Chest X-ray^a^	X	X	X
Ease of Use Questionnaire (study doctor and nurses)	X	X	X
EORTC-QLQ30 and LC13 (patients)^a^	X	X	X
Satisfaction Questionnaire (nurses)	X	X	X
Adverse Event Assessment^a,b^		X	X

^a^Performed during patients' routine medical assessment. ^b^Adverse events were documented and addressed throughout the 42-day study period, not just at scheduled visits.

## Appendix

See Table 3.

## Abbreviations

BDI: Baseline Dyspnea Index
CARE Clinic: Chronic Ascites and Recurrent Effusion Clinic
ECOG: Eastern Cooperative Oncology Group
EORTC: European Organization for Research and Treatment of Cancer
IPC: Indwelling Pleural Catheter
MPE: Malignant Pleural Effusion
QoL: Quality of Life
RCT: Randomized Control Trials
TDI: Transitional Dyspnea Index
TPC: Tunneled Pleural Catheter.
